# Reference photon dosimetry data: A preliminary study of in‐air off‐axis factor, percentage depth dose, and output factor of the Siemens Primus linear accelerator

**DOI:** 10.1120/jacmp.v4i4.2500

**Published:** 2003-09-01

**Authors:** S. H. Cho, G. S. Ibbott

**Affiliations:** ^1^ Department of Radiation Physics The University of Texas M.D. Anderson Cancer Center 1515 Holcombe Boulevard, Unit 547 Houston Texas 77030

**Keywords:** Primus linear accelerators, output factor, off‐axis factor, percentage depth dose, reference photon dosimetry data

## Abstract

The dosimetric characteristics for modern computer‐controlled linear accelerators with the same make, model, and nominal energy are known to be very similar, as long as the machines are unaltered from the manufacturer's original specifications. In this preliminary study, a quantitative investigation of the similarity in the basic photon dosimetry data from the Siemens Primus linear accelerators at eight different institutions is reported. The output factor, percentage depth dose (PDD), and in‐air off‐axis factor (OAF) for the 6 and 18 MV photon beams measured or verified by the Radiological Physics Center (RPC) were analyzed. The RPC‐measured output factors varied by less than about 2% for each field size. The difference between the maximum and minimum RPC‐verified PDD values at each depth was less than about 3%. The difference between the maximum and minimum RPC‐measured in‐air OAF was no more than 4% at all off‐axis distances considered in this study. These results strongly suggest that it is feasible to establish a reference photon dosimetry data set for each make, model, and nominal energy, universally applicable to those machines unaltered from the manufacturers' original specifications, within a clinically acceptable tolerance (e.g., ∼±2%).

PACS number(s): 87.53.–j, 87.66.–a

## INTRODUCTION

Due to improved machining techniques during manufacturing and the use of computers during operation, the construction and dosimetric characteristics of modern computer‐controlled linear accelerators are generally very reproducible.^1^ This observation seems to be valid at least for photon beams whose dosimetric characteristics are generally less sensitive to minute changes in the machine structure than those for electron beams. In fact, matching of dosimetric characteristics among machines is very common. As more machines are being matched, the question arises of how similar are the machines of the same make, model, and nominal energy, across the institutions, in terms of their dosimetric characteristics. Certainly, the benefits from quantifying such similarity would be significant and will be discussed in detail in this article.

One way to perform a systematic and quantitative analysis of the similarity among machines is to investigate some standard behavior among the basic dosimetry data that have been acquired by the Radiological Physics Center (RPC) at M.D. Anderson Cancer Center, through its on‐site dosimetry review program. To demonstrate the efficacy and validity of this approach, a preliminary study was conducted by analyzing three important photon dosimetry data sets, output factor, in‐air off‐axis factor, and percentage depth dose, for one of the recent model linear accelerators, the Siemens Primus (first released in 1997).

**Table I:** Makes and models of the chambers used.

Ion chamber	Wall material	Cavity radius (cm)
NEL 2571	Graphite	0.315
PTW N23333	PMMA	0.305
PTW N30001	PMMA	0.305
Exradin A‐12	C‐552	0.305

## METHODS AND MATERIALS

### Definitions

The definitions of variables and dosimetric quantities used in this study are provided below. Note the following notations are used through out this article, whenever possible:

**rdg**: the electrometer reading following a measurement with an ion chamber.

**CAX**: the central axis of a beam.

**SSD**: the source‐to‐surface distance for a beam. The nominal SSD is 100 cm.

**FS:** the field size of a beam, defined at the nominal SSD.

**RFS**: the reference field size. Note RFS is normally a 10 cm×10 cm field for photon beams.

$d_{\max}$: the depth along the CAX at which the absorbed dose in medium is maximum for a given beam.

$d_{\text{ref}}$: the reference depth along the CAX for measurements in a phantom.

**Fractional Depth Dose (FDD)**: FDD=(rdg at depth on CAX for FS, SSD/rdg at $d_{\max}$ on CAX for FS, SSD)

**Percentage Depth Dose (PDD)**: PDD=FDD×100

**Output Factor (OF):** $\text{OF}=[(\text{rdg}\;\text{at}\;d_{\text{ref}}\;\text{fo}\;\text{FS})/(\text{FDD}\;\text{at}\;d_{\text{ref}}\;\text{for}\;\text{FS})]/[(\text{rdg}\;\text{at}\;d_{\text{ref}}\;\text{for}\;\text{RFS})/(\text{FDD}\;\text{at}\;\text{for}\;\text{RFS})]$

**Off‐Axis Factor (OAF)**: OAF=rdg at off‐axis distance/rdg at CAX

Note OAF is usually defined for the largest field size available (e.g., 40 cm×40 cm for photon beams), at a depth in a phantom or at the isocenter in air.

### Measurements

For the determination of the output factor and depth dose, measurements were performed with Farmer‐type cylindrical chambers in a 30×40×40 cm^3^ water phantom. The makes and models of the chambers used are given in Table I. Identical electrometers (Keithley model 602) were used for measurements. For nonwaterproof chambers, a waterproofing sleeve with a 1 mm thick polymethylmethacrylate (PMMA) wall was used.^2^ Photon beams from the Siemens Primus accelerators were normally incident on the phantom surface at an SSD of 100 cm. The ion chamber readings for depth doses were taken at $d_{\max}$, 5, 10, 15, and 20 cm depths for 6 cm×6 cm, 10 cm×10 cm, and 20 cm×20 cm field sizes. The ion chamber readings for output factors were obtained at a reference depth $\left(d_{\text{ref}}\right)$ of 10 cm for the field sizes, 6 cm×6 cm, 10 cm×10 cm, 15 cm×15 cm, 20 cm×20 cm, and 30 cm×30 cm. The RPC's depths of measurements incorporated a shift to the effective point of measurement following the recommendation from the TG‐51 protocol (i.e., 0.6 times the radius of the chamber cavity upstream from the chamber axis).^2^ At the time of this study, participating institutions measured their PDD data without this shift, however, an appropriate conversion was applied to the RPC‐measured data to enable comparison at the depths reported by the institution (i.e., depths without any shift in the chamber positioning). Consequently, the final results (i.e., PDD and output factor) presented in this article represent measurements at depths with no shift in the chamber positioning.

For the determination of the in‐air off‐axis factor, measurements were performed with either NEL or PTW chambers listed in Table I, at an SAD of 100 cm, with an appropriate plastic buildup cap for the photon energy in question. A 40 cm×40 cm field size was chosen for in‐air measurements. Measurements were made at 5, 10, and 15 cm off‐axis distances from the CAX.

### Data analysis

Eight sets of data, one from each of eight different institutions visited by the RPC were obtained for the 6 and 18 MV photon beams from the Siemens Primus linear accelerators. The dates of first clinical use for these eight machines were evenly spread out over a 3 yr period. Therefore, there was very little correlation between these Primus accelerators, other than the fact that they were unaltered from the manufacturer's original specifications. The institutions' PDDs were verified by the RPC at selected depths (i.e., 5, 10, 15, and 20 cm). The agreement between RPC‐measured and institution's data was better than ±1% for the majority (~90%) of the data points and no worse than ±2% in any case. Although the PDD data for a 30 cm×30 cm field size were not verified by the RPC, a similar agreement was assumed and the PDD data for this field size were included in the analysis. As a result, the final averages as presented in this study were based on the institutions' PDD data to provide a smooth and comprehensive data set over a wide range of depths and field sizes. The RPC does not routinely make measurements in the buildup region; therefore, no PDD data for this region are presented in this study.

All of the 6 MV PDD data were normalized at a depth of 1.5 cm, which is nominally the depth of maximum dose ($d_{\max}$) for the 6 MV photon beam, regardless of the field size. On the other hand, for the 18 MV photon beam, the institutions' PDD data were normalized at different depths depending on the field size. No renormalization of the institution data was attempted during the analysis; instead, the data were accepted as presented by the institutions. A nominal $d_{\max}$ for the 18 MV photon beam is 3.5 cm for a 10 cm×10 cm field size and the institutions investigated in this study chose a depth between 3.0 and 3.5 cm as their $d_{\max}$ depth for this field size. In any case, the impact from this uncertainty in the depth of normalization is believed to be insignificant, because of a relatively large plateau in the PDD for the 18 MV photon beam.

The analysis of the photon output factors was based on the RPC‐measured output factors only, because the RPC‐measured data consistently covered a wide range of field sizes, providing a reasonable characterization of the field size dependence of photon output factors. The agreement between the RPC‐measured and institution's data was as good as that for the PDDs.

Similar to other dosimetry data, eight sets of the RPC‐measured in‐air off‐axis factors were statistically analyzed. Although the beam profiles measured in water are more clinically relevant, in‐air beam profiles could be very helpful in determining the characteristics of the electron pencil beam incident on the target in the head of a linear accelerator^3^ and, therefore, were included in this study.

## RESULTS

The results are presented here as tables so that the data can be easily compared with clinical data. All results are the averages over eight sets of either the RPC‐measured or the RPC‐verified institution data.

### Output factor

Tables II and III show the results of the RPC‐measured output factors for the 6 and 18 MV photon beams, respectively. In addition to the standard deviation, the ratios between the maximum and minimum data are also presented. As seen in these tables, the presented average values are statistically very tight with a maximum standard deviation of about ±0.7% (or max/min ratios less than 1.02), suggesting a possibility for them to be used as reference (or benchmark) data for the Primus linear accelerators.

**Table II:** The RPC‐measured output factors for the 6 MV photon beam from Siemens Primus accelerators. Note the averages were obtained from eight sets of measured data.

Field size	Average OF	Max/min	%SD
6×6	0.954	1.015	0.48
10×10	1.000	‐	‐
15×15	1.034	1.012	0.41
20×20	1.053	1.014	0.45
30×30	1.075	1.020	0.65

### Percentage depth dose

The results from the analysis of the PDD in water are presented in Tables IV and V. Note that each depth indicated in these tables is considered to be the depth without any shift in chamber positioning, as explained earlier. As shown in the tables, the results are again statistically very tight with a maximum standard deviation of about ±1.4% (or max/min ratio always less than 1.03). Consequently, the PDD data presented here could be used as reference data for the Primus accelerators as well.

### In‐air off‐axis factor

The results from the analysis of in‐air OAF are presented in Tables VI and VII. As shown in these tables, the difference between the maximum and minimum values at each off‐axis point was no more than 4%. The largest standard deviation associated with the data was about ±1.3% indicating that in‐air OAF data presented here could be used as reference data for the Primus accelerators. Note the results presented in these tables in‐air OAF and, therefore, may be different from in‐water OAF.

## DISCUSSION

This study shows that some standard behavior among linear accelerators with the same make, model, and nominal energy can be quantitatively investigated. The results strongly suggest that each photon dosimetry characteristic studied (i.e., PDD, output factor, in‐air OAF) could be predicted within a statistical uncertainty comparable to experimental uncertainty. Consequently, it appears possible to establish a basic photon dosimetry data set for each make, model, and nominal energy, universally applicable to those machines unaltered from the manufacturers' original specifications, within a clinically acceptable tolerance (e.g., ∼±2%). If such data sets, referred to as “reference photon dosimetry data” should become available, some potential benefits for the practice of medical physics will be easily seen.

**Table III:** The RPC‐measured output factors for the 18 MV photon beam from Siemens Primus accelerators. Note the averages were obtained from eight sets of measured data.

Field size	Average of	Max/min	%SD
6×6	0.952	1.006	0.22
10×10	1.000	‐	‐
15×15	1.036	1.021	0.69
20×20	1.059	1.018	0.59
30×30	1.080	1.015	0.48

**Table IV:** PDD table for the 6 MV photon beam from Siemens Primus accelerators. Note: (i) the averages were obtained from 8 sets of the RPC‐verified institution data; (ii) max/min values for each average in this table are less than 1.03.

	6 cm×6 cm	10 cm×10 cm		20 cm×20 cm			30 cm×30 cm
Depth (cm)	PDD	%SD	PDD	%SD	PDD	%SD	PDD	%SD
1.5	100.0	‐	100.0	‐	100.0	‐	100.0	‐
2	99.3	0.30	99.1	0.29	99.1	0.21	98.8	0.26
3	95.0	0.24	95.2	0.42	95.5	0.32	95.5	0.37
4	90.3	0.43	91.1	0.45	91.7	0.34	91.8	0.48
5	85.8	0.34	86.9	0.47	88.3	0.42	88.5	0.41
6	81.2	0.21	82.7	0.50	84.3	0.39	85.1	0.40
7	76.8	0.24	78.7	0.38	80.7	0.49	81.6	0.48
8	72.5	0.42	74.6	0.34	77.1	0.52	78.3	0.46
9	68.4	0.54	70.9	0.49	73.6	0.59	74.8	0.79
10	64.8	0.45	67.3	0.61	70.5	0.56	71.8	0.68
15	48.3	0.72	51.3	0.69	55.4	0.76	57.4	0.66
20	36.1	0.84	38.9	0.93	43.2	0.96	45.3	0.78
25	27.0	0.91	29.3	0.67	33.5	0.60	35.5	0.95
30	20.4	0.85	22.4	1.17	26.0	1.16	28.0	0.55

Clearly, the most significant benefit of reference photon dosimetry data would be the ease of the beam commissioning process, especially for photon beams. Note that it is unclear at this time whether or not the approach presented in this study can be successfully applied to electron beams. Normally, medical physicists have to spend several weeks taking beam data during the commissioning of a new machine. Once the reference photon dosimetry data become available, it is conceivable that medical physicists may perform a spot check against a reference photon dosimetry data set for the machine in question, instead of very time‐consuming data taking. Also, it would be possible to envision that generic photon beam models could be constructed using the reference data for the application of convolution or Monte Carlo‐based algorithms so that the commissioning of treatment planning systems could become more standardized and straightforward. At this time, however, it is uncertain whether or not this kind of practice might be allowed in reality, due to various reasons such as possible regulatory concerns. Furthermore, a consensus among medical physicists must be reached in order to allow such practice, even after well‐established reference data become available. Nevertheless, there would be no question about the value of the reference data, especially as a powerful tool for checking the integrity of commissioned data and corresponding beam models.

**Table V:** PDD table for the 18 MV photon beam from Siemens Primus accelerators. Note: (i) the averages were obtained from eight sets of the RPC‐verified institution data; (ii) Note max/min values for each average in this table are less than 1.03; (iii) Atypical $d_{\max}$ at a 10 cm×10 cm field size for 18 MV photon beam is 3.5 cm.

	6 cm×6 cm		10 cm×10 cm		20 cm×20 cm		30 cm×30 cm	
Depth (cm)	PDD	%SD	PDD	%SD	PDD	%SD	PDD	%SD
$d_{\max}$	100.0	‐	100.0	‐	100.0	‐	100.0	‐
4	98.9	0.23	98.8	0.22	98.3	0.34	97.5	0.95
5	95.8	0.49	95.7	0.42	94.9	0.52	94.6	0.49
6	91.8	0.50	92.0	0.39	91.7	0.53	91.2	0.81
7	88.0	0.48	88.3	0.45	88.3	0.47	88.0	0.75
8	84.3	0.57	84.9	0.46	85.1	0.49	84.9	0.73
9	80.7	0.55	81.5	0.44	81.9	0.39	81.9	0.72
10	77.3	0.54	78.1	0.39	78.8	0.58	79.1	0.72
15	61.9	0.65	63.4	0.51	64.9	0.61	65.6	0.59
20	49.6	0.67	51.2	0.57	53.4	0.80	54.4	0.49
25	39.8	0.96	41.5	0.92	43.8	0.83	44.8	1.11
30	32.1	1.12	33.7	1.12	35.9	1.38	37.1	0.61

**Table VI:** In‐air off‐axis factors for the 6 MV photon beam from Siemens Primus accelerators. Note: (i) The averages were obtained from 8 sets of the RPC‐measured data; (ii) The presented values may be different from in‐water OAF.

Off‐axis distance (cm)	Average OAF	Max/min	%SD
5	1.042	1.014	0.42
10	1.058	1.024	0.86
15	1.075	1.037	1.30

Another important benefit from the reference photon dosimetry data could be seen for multi‐institutional quality assurance (QA) monitoring programs such as the Radiological Physics Center (RPC). A previous study^4^ conducted at the RPC suggested that the integrity of clinical dosimetry data as used at each institution participating in national clinical trials could be reviewed by comparing the institutions' data against the RPC's standard data, without visiting each participant to measure its dosimetry data. The major difficulties in performing such a review were the lack of the RPC's standard data for certain dosimerty data (e.g., wedge factor) and some anomalies shown in the RPC's standard data for older machines and electron beams. These difficulties need to be overcome in order for the RPC to successfully utilize this approach. Currently, a major study on this subject is underway at the RPC by applying the Monte Carlo method and new measurement techniques (e.g., ion chamber array, MOSFET, etc.). Electron beams are currently being excluded from this study, because it is still questionable whether or not the institutions' electron dosimetry data can be predicted within an acceptable uncertainty (e.g., ±2%) even by the Monte Carlo method.^5^ In any case, the results (i.e., the reference photon dosimetry data) for most common machines will be presented in the future through peer‐reviewed publications as well as the RPC web site (http://rpc.mdanderson.org), as they become available.

## CONCLUSIONS

The current preliminary study shows that the similarity in the basic photon dosimetry data among linear accelerators with the same make, model, and nominal energy can be quantitatively investigated. A statistical analysis was performed with the basic photon dosimetry data from the Siemens Primus linear accelerators at eight different institutions. The dosimetry data included in the analysis were the output factor, percentage depth dose (PDD), and in‐air off‐axis factor (OAF) for the 6 and 18 MV photon beams, which were measured or verified by the Radiological Physics Center (RPC) through its on‐site dosimetry review program. The RPC‐measured output factors varied by less than about 2% for each field size. The difference between the maximum and minimum RPC‐verified PDD values at each depth was less than about 3%. The difference between the maximum and minimum RPC‐measured in‐air OAF was no more than 4% at all off‐axis distances considered in this study. These results strongly suggest that it is feasible to establish a reference photon dosimetry data set for each make, model, and nominal energy, universally applicable to those machines unaltered from the manufacturers' original specifications, within a clinically acceptable tolerance (e.g., ∼±2%).

**Table VII:** In‐air off‐axis factors for the 18 MV photon beam from Siemens Primus accelerators. Note: (i) The averages were obtained from 8 sets of the RPC‐measured data; (ii) The presented values may be different from in‐water OAF.

Off‐axis distance (cm)	Average OAF	Max/min	%SD
5	1.054	1.033	1.01
10	1.060	1.035	1.12
15	1.063	1.040	1.32

## ACKNOWLEDGMENTS

This investigation was supported by Public Health Service Grant No. CA 10953 awarded by the National Cancer Institute, Department of Health and Human Services. The authors acknowledge the contribution of other physicists at the RPC to the data used in this study. Also, the authors would like to thank Mr. Paul Holguin at the RPC for his help in retrieving the measured data from the RPC database.
